# A comprehensive phenome wide analysis of the role of neutrophils in health and disease

**DOI:** 10.1093/jleuko/qiaf076

**Published:** 2025-05-28

**Authors:** Katy Fleming, Naomi Cornish, Emma E Vincent, Andrew D Mumford, Borko Amulic, Kate Burley

**Affiliations:** School of Cellular and Molecular Medicine, University of Bristol, Biomedical Sciences Building, University Walk, Bristol BS8 1TD, United Kingdom; School of Cellular and Molecular Medicine, University of Bristol, Biomedical Sciences Building, University Walk, Bristol BS8 1TD, United Kingdom; Translational Health Sciences, Bristol Medical School, University of Bristol, Dorothy Hodgkin Building, Whitson Street, Bristol BS1 3NY, United Kingdom; School of Cellular and Molecular Medicine, University of Bristol, Biomedical Sciences Building, University Walk, Bristol BS8 1TD, United Kingdom; School of Cellular and Molecular Medicine, University of Bristol, Biomedical Sciences Building, University Walk, Bristol BS8 1TD, United Kingdom; School of Cellular and Molecular Medicine, University of Bristol, Biomedical Sciences Building, University Walk, Bristol BS8 1TD, United Kingdom

**Keywords:** genome-wide association study, Mendelian randomization, neutrophil, phenome-wide association study

## Abstract

Neutrophil release of cytoplasmic granules containing antimicrobial agents is a critical component of innate immunity. Neutrophils are widely implicated in tissue inflammation however the extent of the neutrophil contribution to human health and disease is incompletely characterized. To explore this further, we leveraged publicly available genetic data to conduct a Mendelian randomization phenome-wide association study (MR-PheWAS) of neutrophil traits and 14,983 outcomes. Genetic proxies for neutrophil count, granularity, and serum myeloperoxidase were linked to 145 outcomes. Higher neutrophil count was associated with lower body weight, reduced obesity risk, and increased vascular activation markers but not with atherosclerosis. Elevated neutrophil count was robustly linked to Alzheimer's disease and neutrophil granularity with gut microbiota abundance and dental pathology. Our findings reveal the diverse roles of neutrophils extending beyond pathogen defense and underscore the potential for MR-PheWAS in identifying novel neutrophil-related pathophysiology.

## Teaser

Neutrophils, essential for immunity, also influence other aspects of health, as shown in our MR-PheWAS study linking genetic proxies for neutrophil traits to 145 outcomes, including obesity, Alzheimer's disease, endothelial activation markers, and gut microbiota.

## 1. Introduction

Neutrophils are the most abundant circulating leucocytes in human blood and function as essential mediators of the innate immune response against invading microorganisms. This function requires recruitment via chemokines to extravascular sites of infection where neutrophils rapidly contain bacterial and fungal proliferation by phagocytosis and the secretion of cytoplasmic granules packed with potent antimicrobial agents, including myeloperoxidase (MPO), lysozyme, and defensins.^1^ In keeping with this critical function, disorders associated with reduced neutrophil number and/or function are associated with immunodeficiency.

There is increasing recognition that neutrophils are also major cellular mediators of inflammation. This activity may contribute to tissue injury responses to infection and to the pathogenesis of autoimmune diseases such as lupus, inflammatory bowel disease, and rheumatoid arthritis.^2–4^ In addition, neutrophils have been implicated in both the development of atherosclerosis and amplification of cardiac injury post–myocardial infarction, with neutrophil count (NEUT) and serum MPO concentration correlating with adverse clinical outcomes.^5–7^

**Fig. 1.**
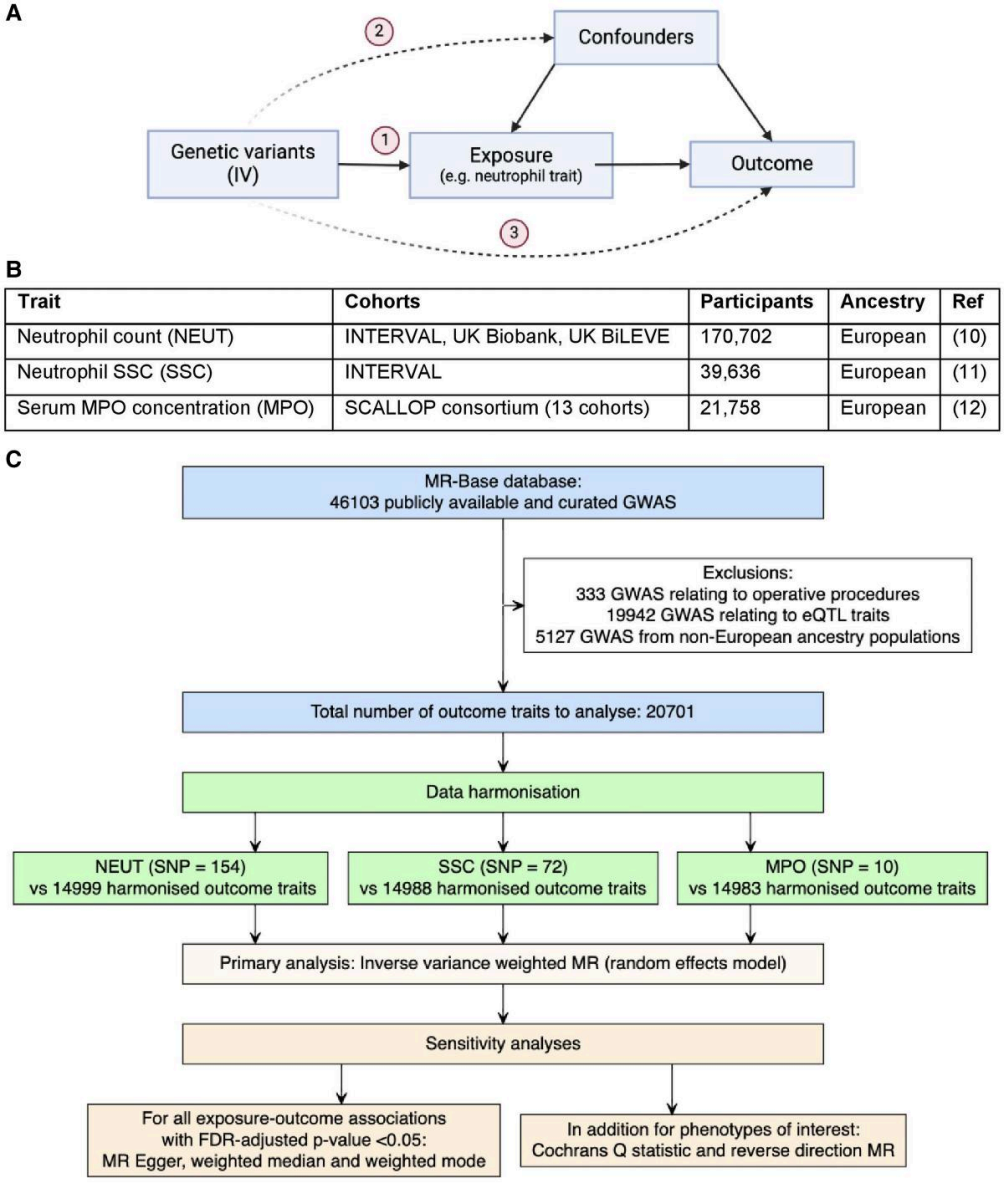
MR-PheWAS methodology. (A) Directed acyclic graph depicting the principle of MR and underlying IV assumptions. Solid arrows indicate causal links and dashed arrows indicate potential violations of MR assumptions.^1–3^ Assumption 1 is that the IV is associated with the exposure of interest (relevance), which can be assessed using the F statistic. Assumption 2 is that the IV is associated with the outcome only through the exposure (independence). With control for population structure in the source GWAS, Mendel's law implies that IVs will not be directly associated with confounders. Assumption 3 is that the IV influences the outcome only through the exposure and not through alternative pathways (exclusion restriction). Primary MR analyses typically assume no pleiotropy, however, sensitivity analyses such as MR Egger calculate the effect estimate adjusted for any directional pleiotropy. (B) Details of GWAS from which exposure instruments were constructed. (C) MR-PheWAS workflow. Three separate PheWASs were undertaken to examine the causal effect of neutrophil traits (exposures) on a range of outcomes. IVs for the exposures were selected, data for these variants were extracted from outcome GWAS summary statistics and then harmonized to ensure that variant effects on the exposure and outcome corresponded to the same allele. Two-sample MR was undertaken, and results interrogated using a range of sensitivity analyses. All analyses were undertaken using the TwoSampleMR R package. eQTL, expression quantitative trait loci; FDR, false discovery rate.

Many of the reported associations between traits such as NEUT and disease outcomes are observational only, thereby creating uncertainty whether neutrophils are causally implicated in pathology or alterations in neutrophil traits are the consequence of disease. Mendelian randomization (MR) is a well-established instrumental variable analysis that addresses some of the shortcomings of conventional observational studies by using genetic proxies for exposures, such as neutrophil traits, to evaluate causal relationships with disease outcomes.^8^ This technique is based on Mendel's law of independent assortment, which states that alleles are sorted into gametes randomly in relation to other parts of the genome. Genetic variants associated with a phenotype can therefore be used to proxy it in a manner independent from confounding influences such as the environment and other traits, including the outcome. In two-sample MR, summary-level data from genome-wide association studies (GWAS) can be used, providing specific assumptions are met, to estimate causal effects (Fig. 1A). MR can then be repeated across numerous outcome traits to systematically scan the phenome for associations with exposures in a hypothesis-free approach.

To better understand the complex role of neutrophils in health and disease, we applied phenome-wide MR analysis (MR phenome-wide association study [MR-PheWAS]) to evaluate the consequences of genetically proxied variation in neutrophil traits on 14,983 outcomes including diseases, anthropometric and behavioral measures, and blood concentrations of biomarkers, proteins, and metabolites. We leveraged GWAS summary data for 3 different neutrophil characteristics: (i) circulating blood NEUT; (ii) neutrophil side scatter (SSC), a measure of intracellular granule content; and (iii) serum concentration of MPO, the most abundant neutrophil granule protein released upon cell activation.^9^

## 2. Materials and methods

### 2.1 Data sources

Analyses were undertaken using data from the largest European-ancestry GWAS for NEUT (n = 170,702) and SSC (n = 39,636) measured as part of the complete blood count test, and MPO (n = 21,758) measured using the Olink proteomics platform (Fig. 1B).^10–12^

### 2.2 Heritability and genetic correlation

Single nucleotide polymorphism (SNP)–based heritability quantifies the extent to which genetic variation, specifically captured by SNPs, contributes to the overall variation observed in a trait. Genetic correlation gives an estimate of the proportion of heritability shared between two complex traits. Both were calculated using LDSC (linkage disequilibrium score regression) software v1.0.1 with European LD scores generated from the 1000 Genomes Project reference panel.^13^

### 2.3 Genetic instruments for neutrophil traits

For NEUT and MPO, all GWAS-significant SNPs (*P* value <5 × 10^−8^) were selected as potential instrumental variables (IVs). SNPs in LD were clumped using an r^2^ threshold of 0.001 (within a 10,000 kB window), resulting in a set of independently associated SNPs for each trait. For SSC, independent SNPs were selected using a multiple stepwise conditional analysis algorithm, as described previously.^11^

### 2.4 Outcome data

Outcome traits were identified using the Medical Research Council Integrative Epidemiology Unit open GWAS database, accessed via the R package TwoSampleMR.^14^ There were 46,103 traits with accessible GWAS summary data on March 11, 2023. Filtering of traits was applied to exclude studies from participants of non-European ancestry, studies of gene expression and administrative codes (such as operative procedures), and any studies missing essential fields required to run MR analysis. This yielded 20,701 outcomes for analysis (Fig. 1C).

### 2.5 MR analysis

Analyses were undertaken using the TwoSampleMR R package. For each neutrophil trait PheWAS, associations for SNPs used as IVs were extracted from summary GWAS data for the filtered outcome phenotypes. If a SNP was not present in the outcome GWAS, data from a proxy SNP in high LD with the target SNP (r^2^ > 0.8) was used, or the SNP removed if no proxies were available. Data were harmonized to ensure the effect of the variant on the outcome and exposure was relative to the same allele. For palindromic SNPs, effect allele frequencies were used to resolve coding strand ambiguities. Steiger filtering was then undertaken to select only those instruments that explained more variance in the exposure than the outcome, thereby minimizing the influence of reverse causality on the analysis. Two-sample MR was conducted for all outcomes with ≥3 SNPs proxying the trait, using multiplicative random effects inverse variance–weighed (IVW) estimates for the primary analysis. Results are expressed as the standard deviation (SD) change (for continuous outcomes) or odds ratio (OR) (for categorical outcomes) per SD increase in genetically predicted neutrophil trait, with corresponding 95% confidence intervals (CIs).

### 2.6 Sensitivity analyses

MR *P* values were adjusted for multiple testing using the false discovery rate correction. Effect estimates using MR methods robust to pleiotropy (MR Egger, weighted median, and weighted mode) were examined, and Cochran's Q statistic was used to test for heterogeneity in genetic instruments. MR analysis was also run in the reverse direction to examine the direction of causality for key findings.

## 2.7 Ethical approval

This analysis used GWAS summary data only, and therefore no additional ethical approval was required.

## 3. Results and discussion

**Fig. 2.**
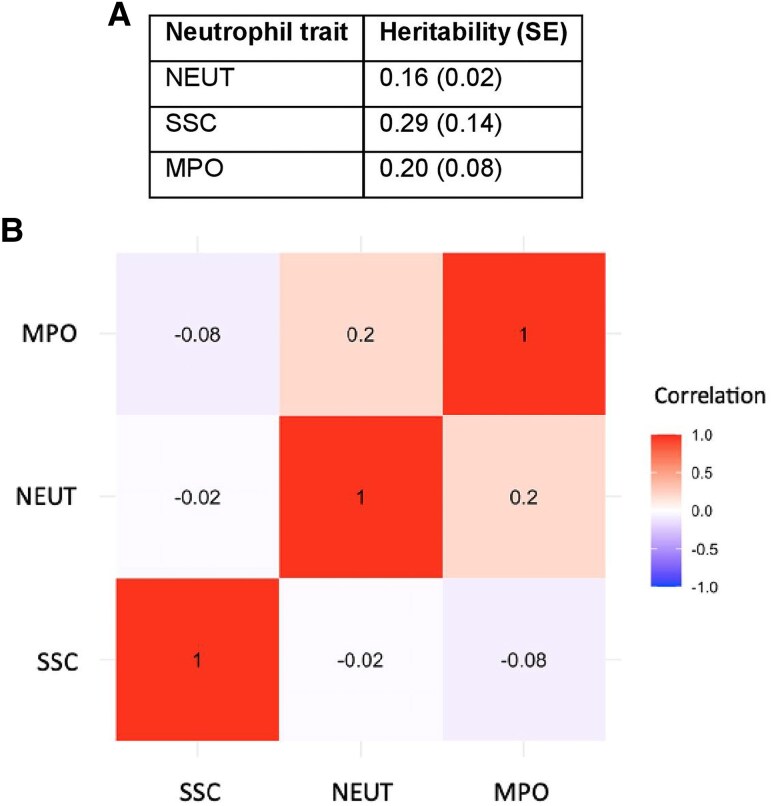
Heritability and genetic correlations for neutrophil traits. (A) Heritability for each neutrophil trait calculated using linkage disequilibrium score regression. (B) Genetic correlation between neutrophil traits. Genetic correlations (rg) calculated using bivariate linkage disequilibrium score regression.

We selected NEUT, SSC, and MPO as exposure traits because they are relevant to neutrophil biology and can be measured using high-throughput laboratory analyses of large population collections, thereby enabling robust genetic proxies to be determined by GWAS. SNP-based heritability for all 3 exposures were comparable to values reported for other blood cell traits (Fig. 2A).^10^

Using independently associated SNPs from the largest published GWAS for each trait, NEUT, SSC, and MPO were genetically proxied by 154, 72, and 10 variants, respectively. The mean F statistic, an estimate of the strength of each instrument-exposure association, was 74.7 for NEUT, 247.1 for SSC, and 89.2 for MPO, thereby exceeding the widely reported threshold of >10 and indicating minimal risk of weak instrument bias for MR analyses.^15^

**Fig. 3.**
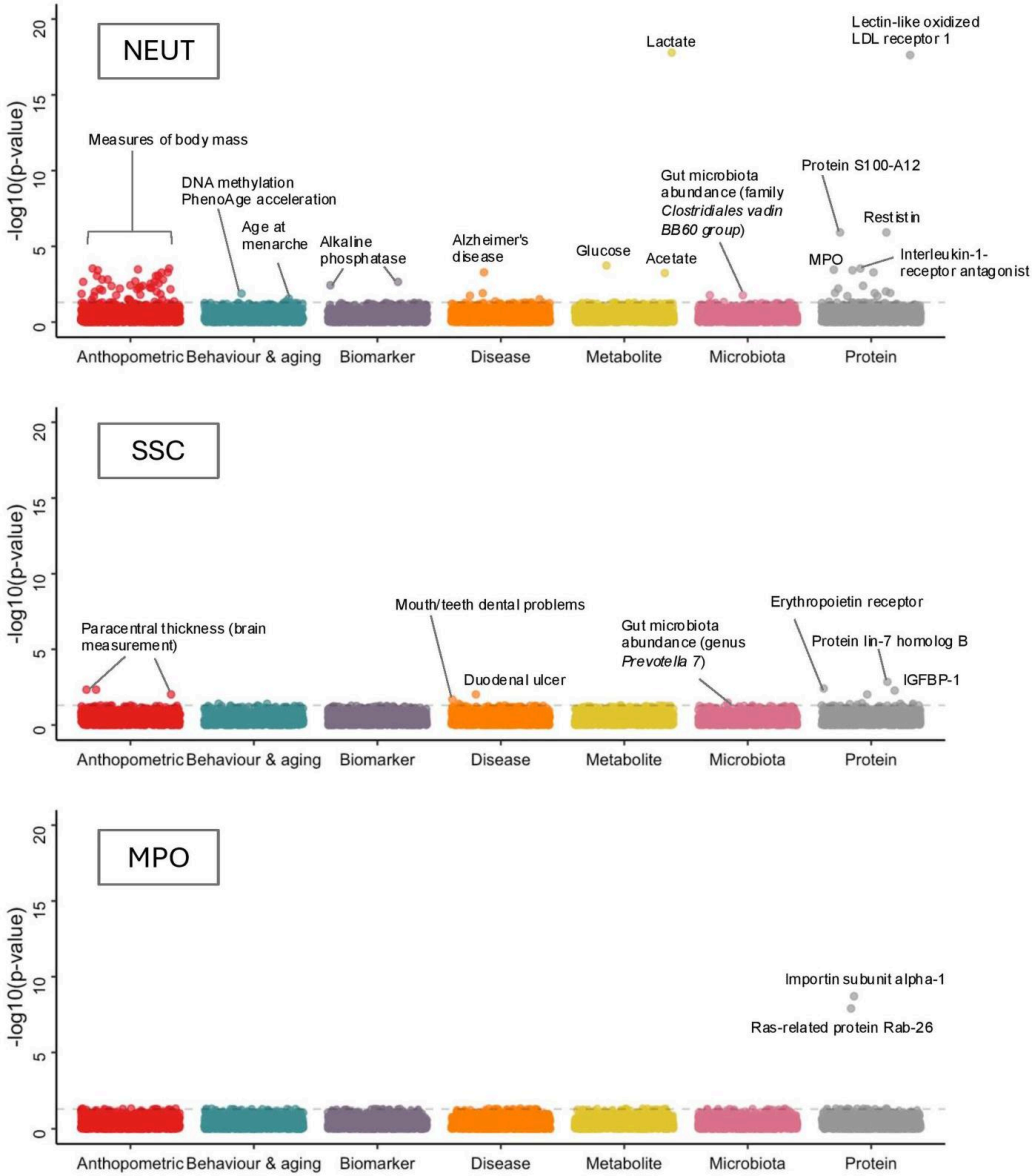
Phenome-wide associations with neutrophil characteristics. Manhattan plots for NEUT, SSC, and MPO. Hematological parameters excluded to aid visualization. The dashed gray line indicates false discovery rate–adjusted *P* value of 0.05.

MR-PheWAS demonstrated that neutrophil traits had a genetically predicted effect on a total of 145 outcomes (false discovery rate–adjusted *P* < 0.05) (Tables S1, S2, and S3). Reflecting the number of SNPs used to instrument each trait, NEUT had the greatest number of associations (117), followed by SSC (26) and MPO (2) (Fig. 3; Fig. S1).

### 3.1 Relationship between NEUT and blood cell phenotypes

Of the 117 associations with NEUT, 40 related to other blood cell parameters. These relationships were likely to be reflecting shared genetic architecture and/or polygenicity-induced horizontal pleiotropy, and therefore were not explored in depth. However, we note a positive correlation between NEUT and quantification of all other blood cells, including red blood cell count. As neutrophilia is a widely reported marker of inflammation, this suggests that NEUT in population cohorts reflects increased hematopoiesis rather than chronic inflammatory states, typically characterized by anemia. In keeping with this, genetically predicted increasing NEUT associated with higher serum alkaline phosphatase (SD 0.09 [95% CI 0.05, 0.13]; adjusted *P* = 2.21 × 10^−3^), a measure of bone turnover, and there was little causal evidence for any of the neutrophil traits in this study on biomarkers of inflammation such as C-reactive protein (adjusted *P* > 0.05).

### 3.2 Neutrophil traits associate with plasma protein levels

The traits NEUT and MPO were noted to have a weak positive genetic correlation (rg 0.2), in keeping with previous functional studies (Fig. 2B).^16,17^ Consistent with this, MR analysis identified an association between NEUT and MPO as well as six other constituents of the neutrophil releasate including bactericidal permeability-increasing protein (UniProt P17213; SD 0.30 [95% CI 0.14, 0.44]; adjusted *P =* 1.92 × 10^−2^) and lactotransferrin (UniProt P02788; SD 0.32 [95% CI 0.16, 0.47]; adjusted *P =* 9.49 × 10^−3^). This supports the hypothesis that neutrophils have a basal release of neutrophil granules, and therefore subjects with increased NEUTs have higher serum concentrations of granule markers.^18^

SSC was not genetically correlated with either NEUT or MPO (rg −0.02 and −0.08, respectively), indicating that these are discrete cellular phenotypes. Both SSC and MPO associated with the serum concentrations of proteins implicated in intracellular membrane trafficking, including glycolipid transfer protein (UniProt Q9NZD2; SD −0.12 [95% CI −0.18, −0.07]; adjusted *P =* 9.54 × 10^−3^), polyphosphoinositide phosphatase (UniProt Q92562; SD −0.11 [95% CI −0.17, −0.06]; adjusted *P =* 3.64 × 10^−3^), and Ras-related protein Rab-26 (UniProt Q9ULW5; SD 0.89 [95% CI 0.64, 1.15]; adjusted *P =* 1.25 × 10^−8^).

### 3.3 NEUT associates with lower body mass

**Fig. 4.**
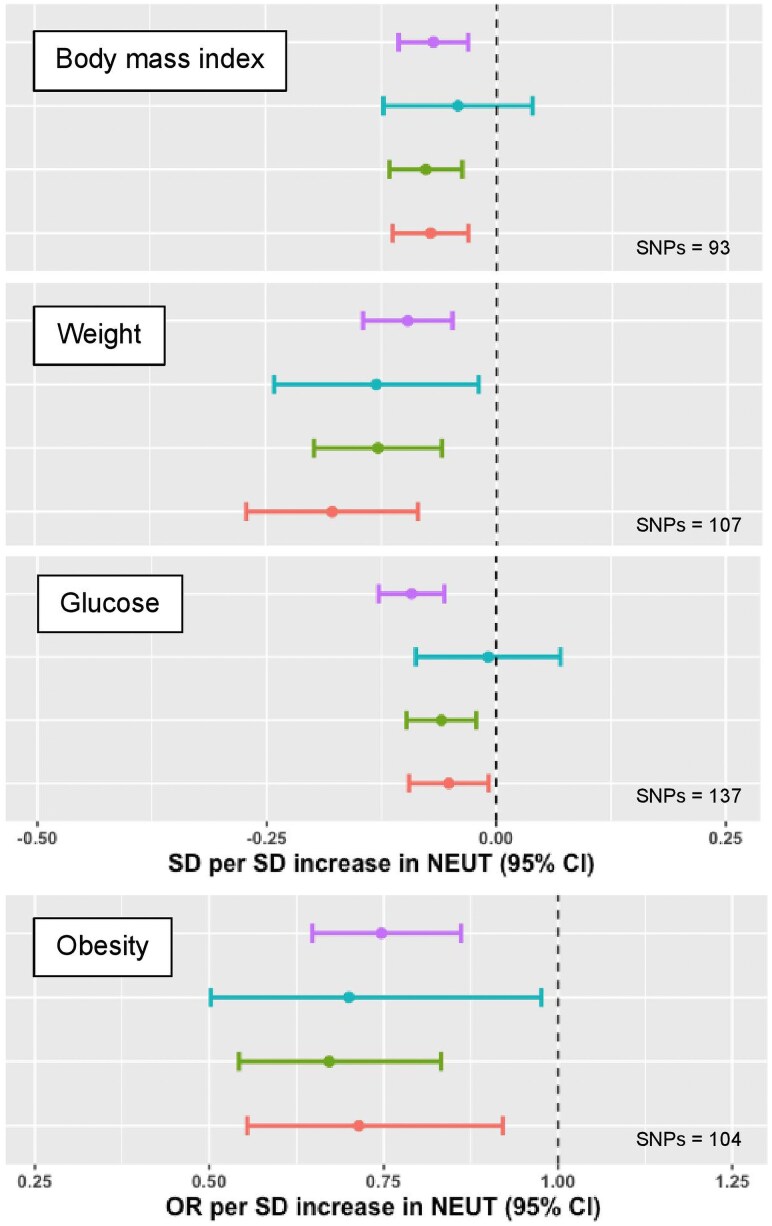
MR of NEUT associations with body mass. Forest plots with MR effect estimates for body mass index, weight, glucose, and obesity. IVW (primary analysis) is shown in in purple, MR Egger is shown in blue, the weighted median is shown in green, and the weighted mode is shown in red. Error bars signify 95% CIs.

Higher NEUT was causally associated with reduced body weight (SD −0.10 [95% CI −0.14, −0.05]; adjusted *P* = 1.57 × 10^−2^), reduced body mass index (SD −0.07 [95% CI −0.10, −0.03]; adjusted *P* = 4.60 × 10^−2^), and lower risk of obesity (OR 0.75 [95% CI 0.65, 0.86]; adjusted *P* = 9.85 × 10^−3^) (Fig. 4). Pleiotropy robust MR methods showed concordant effect sizes and no reverse associations between body weight, body mass index, and obesity with NEUT as the outcome (Table S4). NEUT also showed associations with impedance-derived anthropometric traits such as reduced trunk, arm, and leg predicted and fat-free mass, although there was evidence of confounding by reverse causality for these traits. Cochran's Q statistics suggested heterogeneity between the SNPs used to instrument all measures of body mass, indicating potential pleiotropic pathways (Table S5). These findings may be indicative of a more complex relationship between neutrophils and body mass, as prior observations have indicated that circulating NEUTs are elevated in obesity and that this corrects in response to weight loss after bariatric surgery.^19,20^

We identified several potential mechanisms by which neutrophils may reduce body mass. For example, increased NEUT was associated with lower serum glucose (SD −0.09 [95% CI −0.13, −0.06]; adjusted *P* = 1.84 × 10^−4^). Increased SSC associated with increased serum concentrations of both insulin-like growth factor-binding protein 1 (UniProt P08833; SD 0.38 [95% CI 0.22, 0.53]; adjusted *P* = 5.28 × 10^−3^), and angiopoietin-related protein 3 (UniProt Q9Y5C1; SD 0.12 [95% CI 0.06, 0.18]; adjusted *P* = 4.18 × 10^−2^), which are both implicated in regulating insulin sensitivity and have previously been inversely associated with obesity and metabolic syndrome, although no associations between SCC and measures of body mass were identified in our study.^21–24^ Together, these findings suggest that neutrophils may influence body mass by altering glucose metabolism.

### 3.4 NEUT associates with endothelial activation but not with vascular disease

Increased NEUT associated with increased serum E-selectin (UniProt P16581; SD 0.13 [95% CI 0.08, 0.19]; adjusted *P* = 5.21 × 10^−4^) and oxidized low-density lipoprotein receptor 1 (UniProt P78380; SD 0.32 [95% CI 0.16, 0.48]; adjusted *P* = 1.20 × 10^−2^), both of which are secreted by the vascular endothelium in response to inflammatory stimuli. This is in keeping with experimental findings that neutrophils promote endothelial activation.^25^

However, despite this association, we found little evidence for a causal relationship between any of the neutrophil traits and vascular pathologies such as hypertension, atherosclerosis, myocardial infarction, peripheral vascular disease, or stroke (adjusted *P* > 0.05). Multiple previous observational studies have shown that higher NEUTs are associated with increased risk of coronary heart disease.^26,27^ However, this finding was not replicated in our study, which accords with previous MR analyses.^10,28^ This indicates potential confounding in the prior observational studies and highlights that genetic effects on intermediate phenotypes such as molecular pathways can be diluted or opposed downstream before exerting an influence on complex pathologies such as vascular disease.

### 3.5 Neutrophil traits associate with alterations in the gut microbiome

**Fig. 5.**
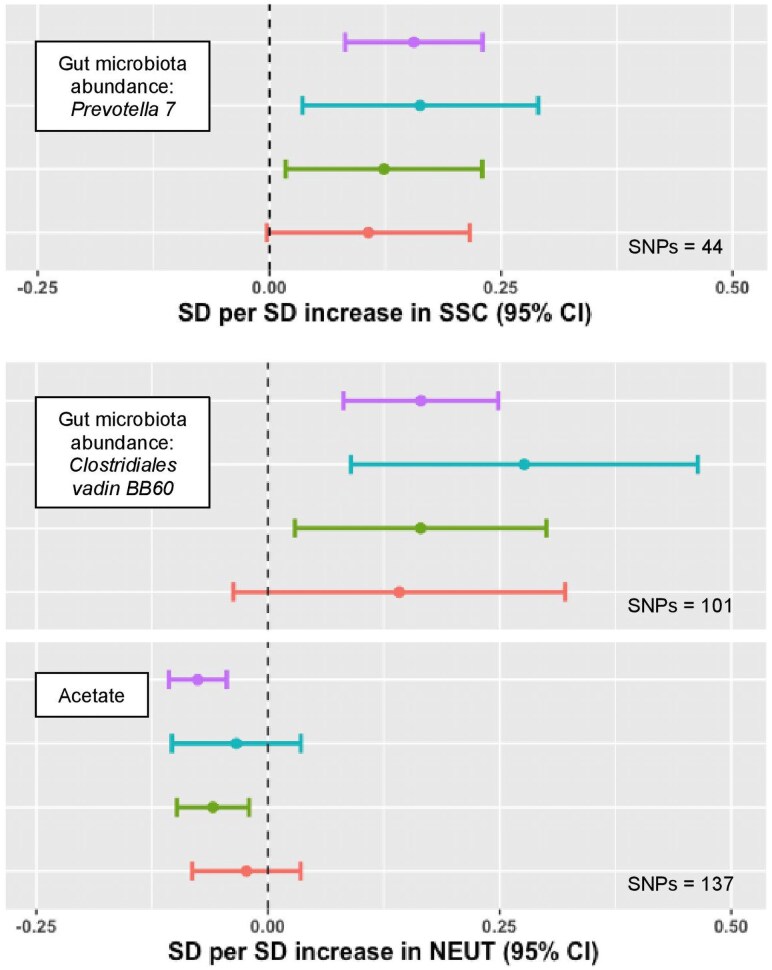
MR of neutrophil trait associations with the gut microbiome. Forest plots with MR effect estimates for abundance of gut microbiota and serum acetate. IVW (primary analysis) is shown in in purple, MR Egger is shown in blue, the weighted median is shown in green, and the weighted mode is shown in red. Error bars signify 95% confidence intervals.

MR analyses found that neutrophil traits were causally associated with alterations in the gut microbiome. Increased NEUT associated with the abundance of *Clostridiales vadin BB60* group bacteria (SD 0.17 [95% CI 0.08, 0.25]; adjusted *P* = 1.69 × 10^−2^) and increased neutrophil SSC associated with abundance of genus *Prevotella* 7 (SD 0.16 [95% CI 0.08, 0.23]; adjusted *P* = 3.35 × 10^−2^), both short-chain fatty acid–producing bacteria (Fig. 5). Interestingly, NEUT was associated with reduced serum levels of acetate (SD −0.08 [95% CI −0.11, −0.04]; adjusted *P* = 5.79 × 10^−4^), the most abundant gut-derived short-chain fatty acid in the systemic circulation. This aligns with findings in murine models where colonization of mice with *Prevotella* counterintuitively results in a reduction of acetate, exacerbating intestinal inflammation.^29^

These findings are in keeping with the established role of neutrophils in regulating the composition of gut commensal species, with neutrophil dysfunction implicated in dysbiosis, an imbalance in microbial populations, which can promote disease states.^30,31^ Abundance of *C. vadin BB60* has been inversely associated with alterations in insulin resistance and obesity in previous observational studies, suggesting a further mechanism through which neutrophils may influence body mass.^32^ SSC was also associated with dental problems (OR 0.99 [95% CI 0.99, 0.99]; adjusted *P* = 3.95 × 10^−2^) and duodenal ulcer (OR 0.99 [95% CI 0.99, 0.99]; adjusted *P* = 9.54 × 10^−3^), both of which have been linked to alterations in gut microbiota, including *Prevotella*, in other studies.^33,34^ Interestingly, these phenotypes accord with the observation that patients with disorders of neutrophil number and/or function are prone to developing periodontitis and mucosal ulceration.^35,36^

### 3.6 NEUT associated with risk of Alzheimer's disease

**Fig. 6.**
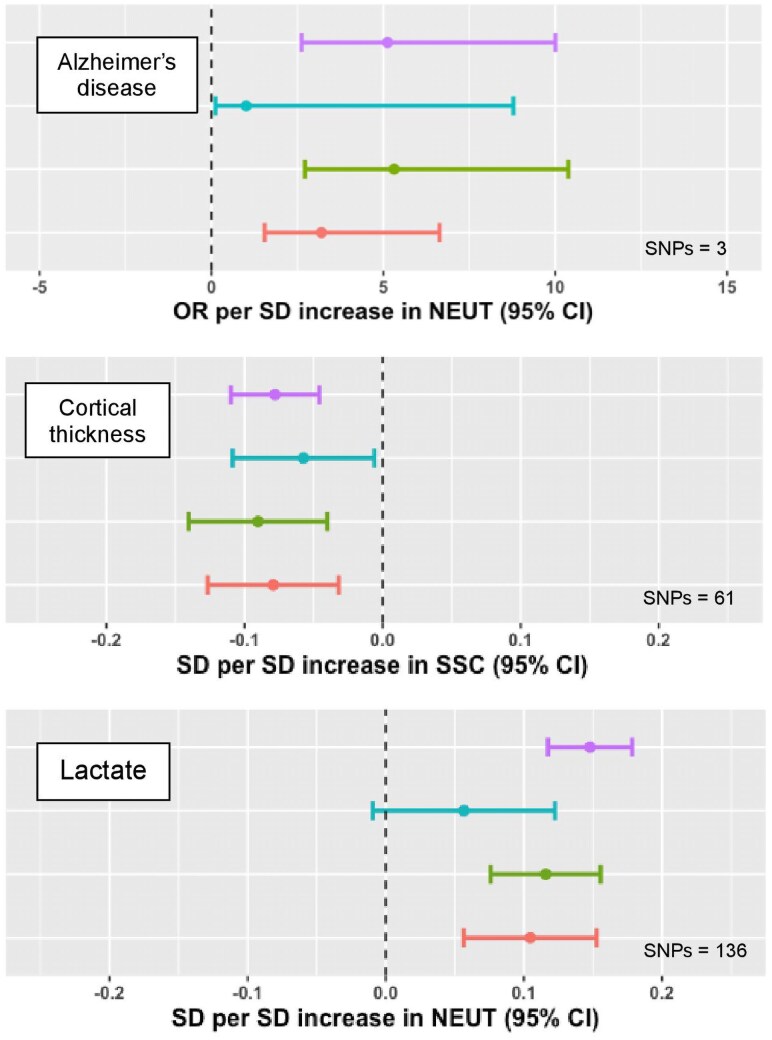
MR of neutrophil trait associations with AD. Forest plots with MR effect estimates for AD, brain cortical thickness, and serum lactate. IVW (primary analysis) is shown in in purple, MR Egger is shown in blue, the weighted median is shown in green, and the weighted mode is shown in red. Error bars signify 95% CIs.

We found that increased NEUT associated with an increased risk of Alzheimer's disease (AD) (OR 5.12 [95% CI 2.62, 10.01]; adjusted *P* = 5.21 × 10^−4^) (Fig. 6). Pleiotropy robust MR estimates were concordant, Cochran's Q statistic indicated homogeneity of IVs and MR in the reverse direction showed no significant associations, although we note that this association was proxied by only 3 SNPs. Increased SSC was associated with reduced measurements of cortical thickness in the brain (SD −0.08 [95% CI −0.10, −0.05]; adjusted *P* = 4.63 × 10^−3^), which is a proposed biomarker of neurodegeneration, correlating with cognitive impairment and development of AD.^37,38^

A possible role for neutrophils in the pathogenesis of AD has been suggested as neutrophil infiltration exacerbates neuroinflammation in animal AD models and neutrophil depletion or inhibition of recruitment reduces the formation of AD-associated amyloid-beta plaques.^39^ Consistent with our findings, observational studies have linked NEUT to AD risk and reduced cognitive function.^40,41^ It is noteworthy that we also found that NEUT associated with blood lactate concentration (SD 0.15 [95% CI 0.12, 0.18]; adjusted *P* = 1.64 × 10^−18^). Lactate, a metabolite produced by anaerobic metabolism in neutrophils, is a modulator of neuronal function and long-term memory and is increasingly implicated in AD pathogenesis.^42,43^

### 3.7 Study limitations

Although exposure IVs were constructed from the largest GWAS datasets available for the neutrophil traits, the power of our MR-PheWAS is constrained by the sample size of these studies, particularly for serum MPO concentration (n = 21,758). Similarly, some disorders are either absent or only represented by small GWAS in MR-Base, which was used to ascertain outcomes. Associations were tested between individual SNPs and outcomes as opposed to SNPs being weighted and combined in a polygenic risk score for each neutrophil trait, an approach that may reveal additional novel findings. We are aware that there is likely to be some overlap between the exposure and outcome GWAS samples for some of the phenotypes, many of which included UK Biobank participants. This may potentially introduce bias leading to inflated type I error rates.^44^ Finally, we acknowledge that our analysis was conducted using data from individuals of European ancestry and thus the results may not be generalizable to other populations.

## 4. Conclusions

Here, we present the first comprehensive MR-PheWAS for neutrophil traits and provide evidence for causal associations between neutrophil traits and outcomes relating to body mass, endothelial activation, the gut microbiome, and AD. Hypotheses generated in this study now require orthogonal confirmation from observational population data and focused functional studies.

## Supplementary Material

qiaf076_Supplementary_Data

## Data Availability

R scripts used for the analysis are available via GitHub (https://github.com/kateburley/Neutrophil_PheWAS). Harmonized summary data for all SNPs included in this analysis and full MR results are available through the University of Bristol Research Data Repository.

## References

[1] Lehman HK, Segal BH. The role of neutrophils in host defense and disease. J Allergy Clin Immunol. 2020:145:1535–1544. 10.1016/j.jaci.2020.02.038 32283205 PMC8912989

[2] Liu Y, Kaplan MJ. Neutrophil dysregulation in the pathogenesis of systemic lupus erythematosus. Rheum Dis Clin North Am. 2021:47:317–333. 10.1016/j.rdc.2021.04.002 34215366

[3] Soderberg D, Segelmark M. Neutrophil extracellular traps in vasculitis, friend or foe? Curr Opin Rheumatol. 2018:30:16–23. 10.1097/BOR.0000000000000450 28957962

[4] Fresneda Alarcon M, McLaren Z, Wright HL. Neutrophils in the pathogenesis of rheumatoid arthritis and systemic lupus erythematosus: same foe different M.O. Front Immunol. 2021:12:649693. 10.3389/fimmu.2021.649693 33746988 PMC7969658

[5] Luo J, Thomassen JQ, Nordestgaard BG, Tybjaerg-Hansen A, Frikke-Schmidt R. Neutrophil counts and cardiovascular disease. Eur Heart J. 2023:44:4953–4964. 10.1093/eurheartj/ehad649 37950632 PMC10719495

[6] Balta S, et al The relation between atherosclerosis and the neutrophil-lymphocyte ratio. Clin Appl Thromb Hemost. 2016:22:405–411. 10.1177/1076029615569568 25667237

[7] Zhang N, et al Correlation analysis of plasma myeloperoxidase level with global registry of acute coronary events score and prognosis in patients with acute non-ST-segment elevation myocardial infarction. Front Med (Lausanne). 2022:9:828174. 10.3389/fmed.2022.828174 35419382 PMC8995496

[8] Hemani G, Tilling K, Smith GD. Orienting the causal relationship between imprecisely measured traits using GWAS summary data. PLoS Genet. 2017:13:e1007081. 10.1371/journal.pgen.1007081 29149188 PMC5711033

[9] Zimmermann M, Cremer M, Hoffmann C, Weimann K, Weimann A. Granularity Index of the SYSMEX XE-5000 hematology analyzer as a replacement for manual microscopy of toxic granulation neutrophils in patients with inflammatory diseases. Clin Chem Lab Med. 2011:49:1193–1198. 10.1515/CCLM.2011.188 21574880

[10] Astle WJ, et al The allelic landscape of human blood cell trait variation and links to common Complex disease. Cell. 2016:167:1415–1429.e19. 10.1016/j.cell.2016.10.042 27863252 PMC5300907

[11] Akbari P, et al A genome-wide association study of blood cell morphology identifies cellular proteins implicated in disease aetiology. Nat Commun. 2023:14:5023. 10.1038/s41467-023-40679-y 37596262 PMC10439125

[12] Folkersen L, et al Mapping of 79 loci for 83 plasma protein biomarkers in cardiovascular disease. PLoS Genet. 2017:13:e1006706. 10.1371/journal.pgen.1006706 28369058 PMC5393901

[13] Bulik-Sullivan BK, et al LD score regression distinguishes confounding from polygenicity in genome-wide association studies. Nat Genet. 2015:47:291–295. 10.1038/ng.3211 25642630 PMC4495769

[14] Hemani G, et al The MR-Base platform supports systematic causal inference across the human phenome. Elife. 2018:7:e34408. 10.7554/eLife.34408 29846171 PMC5976434

[15] Sanderson E, Spiller W, Bowden J. Testing and correcting for weak and pleiotropic instruments in two-sample multivariable Mendelian randomization. Stat Med. 2021:40:5434–5452. 10.1002/sim.9133 34338327 PMC9479726

[16] Wang Y, et al Neutrophil extracellular trap burden correlates with the stenosis of coronary atherosclerosis. PeerJ. 2023:11:e15471. 10.7717/peerj.15471 37304868 PMC10252804

[17] Hoy A, et al Serum myeloperoxidase concentration in a healthy population: biological variations, familial resemblance and new genetic polymorphisms. Eur J Hum Genet. 2001:9:780–786. 10.1038/sj.ejhg.5200702 11781690

[18] Adrover JM, et al Programmed ‘disarming’ of the neutrophil proteome reduces the magnitude of inflammation. Nat Immunol. 2020:21:135–144. 10.1038/s41590-019-0571-2 31932813 PMC7223223

[19] Herishanu Y, Rogowski O, Polliack A, Marilus R. Leukocytosis in obese individuals: possible link in patients with unexplained persistent neutrophilia. Eur J Haematol. 2006:76:516–520. 10.1111/j.1600-0609.2006.00658.x 16696775

[20] Dixon JB, O'Brien PE. Obesity and the white blood cell count: changes with sustained weight loss. Obes Surg. 2006:16:251–257. 10.1381/096089206776116453 16545154

[21] Petersson U, Ostgren CJ, Brudin L, Brismar K, Nilsson PM. Low levels of insulin-like growth-factor-binding protein-1 (IGFBP-1) are prospectively associated with the incidence of type 2 diabetes and impaired glucose tolerance (IGT): the soderakra cardiovascular risk factor study. Diabetes Metab. 2009:35:198–205. 10.1016/j.diabet.2008.11.003 19297224

[22] Fontana L, et al Effects of 2-year calorie restriction on circulating levels of IGF-1, IGF-binding proteins and cortisol in nonobese men and women: a randomized clinical trial. Aging Cell. 2016:15:22–27. 10.1111/acel.12400 26443692 PMC4717266

[23] Cinkajzlova A, et al Angiopoietin-like protein 3 and 4 in obesity, type 2 diabetes mellitus, and malnutrition: the effect of weight reduction and realimentation. Nutr Diabetes. 2018:8:21. 10.1038/s41387-018-0032-2 29695708 PMC5916880

[24] Hezarkhani S, et al The serum levels of angiopoietin-like protein 3 and 4 in type 2 diabetic patients with and without metabolic syndrome compared to the control group. Endocrinol Diabetes Metab. 2024:7:e466. 10.1002/edm2.466 38140923 PMC10782050

[25] Knackstedt SL, et al Neutrophil extracellular traps drive inflammatory pathogenesis in malaria. Sci Immunol. 2019:4:eaaw0336. 10.1126/sciimmunol.aaw0336 31628160 PMC6892640

[26] Baldus S, et al Myeloperoxidase serum levels predict risk in patients with acute coronary syndromes. Circulation. 2003:108:1440–1445. 10.1161/01.CIR.0000090690.67322.51 12952835

[27] Gaul DS, Stein S, Matter CM. Neutrophils in cardiovascular disease. Eur Heart J. 2017:38:1702–1704. 10.1093/eurheartj/ehx244 30052884

[28] Cupido AJ, et al Genetically predicted neutrophil-to-lymphocyte ratio and coronary artery disease: evidence from Mendelian randomization. Circ Genom Precis Med. 2022:15:e003553. 10.1161/CIRCGEN.121.003553 35103484 PMC7612391

[29] Iljazovic A, et al Perturbation of the gut microbiome by Prevotella spp. Enhances host susceptibility to mucosal inflammation. Mucosal Immunol. 2021:14:113–124. 10.1038/s41385-020-0296-4 32433514 PMC7790746

[30] Zhang D, Frenette PS. Cross talk between neutrophils and the microbiota. Blood. 2019:133:2168–2177. 10.1182/blood-2018-11-844555 30898860 PMC6524562

[31] Carding S, Verbeke K, Vipond DT, Corfe BM, Owen LJ. Dysbiosis of the gut microbiota in disease. Microb Ecol Health Dis. 2015:26:26191. 10.3402/mehd.v26.26191 25651997 PMC4315779

[32] Sawicka-Smiarowska E, et al Gut microbiome in chronic coronary syndrome patients. J Clin Med. 2021:10:5074. 10.3390/jcm10215074 34768594 PMC8584954

[33] Wu GD, et al Linking long-term dietary patterns with gut microbial enterotypes. Science. 2011:334:105–108. 10.1126/science.1208344 21885731 PMC3368382

[34] Chen X, et al Comparisons between bacterial communities in Mucosa in patients with gastric antrum ulcer and a duodenal ulcer. Front Cell Infect Microbiol. 2018:8:126. 10.3389/fcimb.2018.00126 29868487 PMC5952031

[35] Ye Y, et al Mutations in the ELANE gene are associated with development of periodontitis in patients with severe congenital neutropenia. J Clin Immunol. 2011:31:936–945. 10.1007/s10875-011-9572-0 21796505 PMC3223588

[36] Guarino AD, et al Cyclic neutropenia mimicking Crohn's disease: two case reports and a narrative review. J Clin Med. 2023:12:6323. 10.3390/jcm12196323 37834967 PMC10573598

[37] Yang H, et al Study of brain morphology change in Alzheimer's disease and amnestic mild cognitive impairment compared with normal controls. Gen Psychiatr. 2019:32:e100005. 10.1136/gpsych-2018-100005 31179429 PMC6551438

[38] Dickerson BC, et al The cortical signature of Alzheimer's disease: regionally specific cortical thinning relates to symptom severity in very mild to mild AD dementia and is detectable in asymptomatic amyloid-positive individuals. Cereb Cortex. 2009:19:497–510. 10.1093/cercor/bhn113 18632739 PMC2638813

[39] Zenaro E, et al Neutrophils promote Alzheimer's disease-like pathology and cognitive decline via LFA-1 integrin. Nat Med. 2015:21:880–886. 10.1038/nm.3913 26214837

[40] Luo J, Thomassen JQ, Nordestgaard BG, Tybjaerg-Hansen A, Frikke-Schmidt R. Blood leukocyte counts in Alzheimer disease. JAMA Netw Open. 2022:5:e2235648. 10.1001/jamanetworkopen.2022.35648 36215071 PMC9552891

[41] Fa W, et al Associations of blood absolute neutrophil count and cytokines with cognitive function in dementia-free participants: a population-based cohort study. J Gerontol A Biol Sci Med Sci. 2024:79:glad231. 10.1093/gerona/glad231 37777477

[42] Wu A, Lee D, Xiong WC. Lactate metabolism, signaling, and function in brain development, synaptic plasticity, angiogenesis, and neurodegenerative diseases. Int J Mol Sci. 2023:24:13398. 10.3390/ijms241713398 37686202 PMC10487923

[43] Liguori C, et al Cerebrospinal fluid lactate levels and brain [18F]FDG PET hypometabolism within the default mode network in Alzheimer's disease. Eur J Nucl Med Mol Imaging. 2016:43:2040–2049. 10.1007/s00259-016-3417-2 27221635

[44] Burgess S, Davies NM, Thompson SG. Bias due to participant overlap in two-sample Mendelian randomization. Genet Epidemiol. 2016:40:597–608. 10.1002/gepi.21998 27625185 PMC5082560

