# Supplementary figures and images for: A comprehensive phenome wide analysis of the role of neutrophils in health and disease
Source: J Leukoc Biol. 2025 May 28;117(7):qiaf076. doi: 10.1093/jleuko/qiaf076 (PMC12257109; doi:10.1093/jleuko/qiaf076)

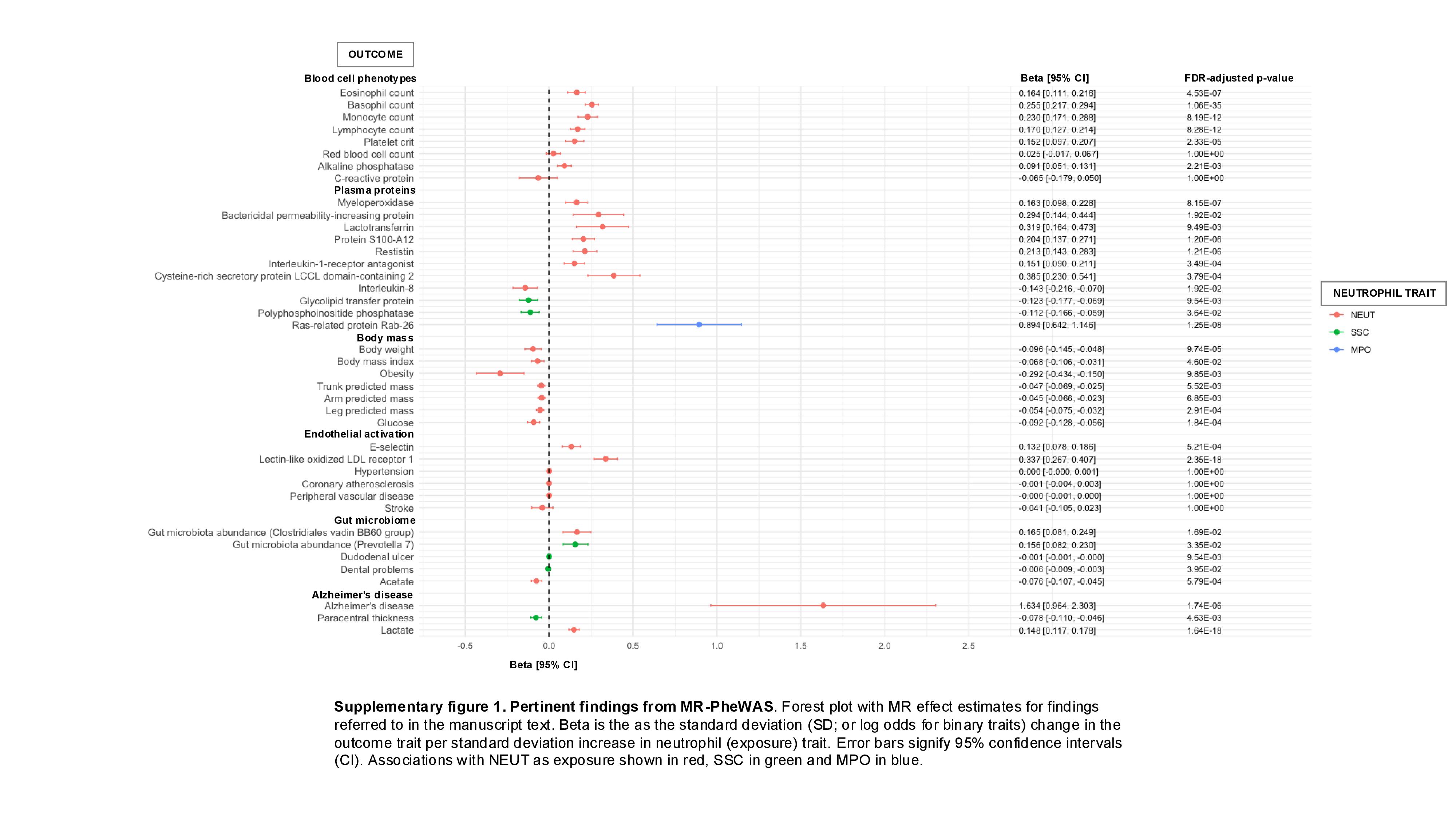

Supplement: qiaf076_Supplementary_Data [file qiaf076_supplementary_data.zip › Supp_figure_1.jpg]
